# Physical and Numerical Investigation of Hot Deformation Behavior in Medium-Mn Steel for Automotive Forgings

**DOI:** 10.3390/ma18081883

**Published:** 2025-04-21

**Authors:** Aleksandra Kozłowska, Sebastian Sławski, Wojciech Borek, Adam Grajcar

**Affiliations:** 1 Department of Engineering Materials and Biomaterials, Faculty of Mechanical Engineering, Silesian University of Technology, 18A Konarskiego Street, 44-100 Gliwice, Poland; aleksandra.kozlowska@polsl.pl (A.K.); wojciech.borek@polsl.pl (W.B.); 2 Department of Theoretical and Applied Mechanics, Faculty of Mechanical Engineering, Silesian University of Technology, 44-100 Gliwice, Poland; sebastian.slawski@polsl.pl

**Keywords:** finite element method, advanced high-strength steel, automotive forging, numerical investigation, Gleeble simulation

## Abstract

In this study, the hot deformation behavior of novel 0.17C-3.92Mn-1.02Si-0.53Al-0.22Mo-0.032Ti-0.069V steel during continuous compression was predicted using numerical simulation, providing a reference for optimizing the process. Medium-Mn steels have not been applied for forgings yet. Therefore, their industrial application requires detailed investigations on their hot deformability. Results of finite element (FEM) simulations will be used for further optimization of the press forging process. The material model parameters used in the FEM method were identified based on stress–strain curves registered during hot compression tests carried out using a Gleeble thermomechanical simulator. The numerical simulation and physical investigations were performed at temperatures of 900, 1000 and 1100 °C to reflect a range of temperatures occurring during press forging. The influence of strain rates of 0.05, 0.5 and 5 s^−1^ on the flow behavior of steel was also investigated. Colored maps of the plastic strain distribution in a sample volume were obtained as a result of the numerical research. The maps allowed for the identification of differently strengthened zones as a result of varied plastic strain. Results of FEM analysis were experimentally validated by hardness measurements. A good correlation between the hardness and plastic deformation zones was obtained. An increase in the material hardness was identified in the zones characterized by the highest plastic strain.

## 1. Introduction

The 42CrMo4 grade steel is widely used for automotive forged components like steering gears [1]. The typical heat treatment process of forgings made of 42CrMo4 type steel consists of quenching and tempering carried out at relatively high temperatures of 400–650 °C [2], which makes this process energy-consuming [3]. The high hardenability and high strength properties of 42CrMo4 type steel are achieved by a Cr addition (1–1.5 wt.%), which is an expensive element, and by elevated carbon content (0.4–0.5 wt.%), which deteriorates the machinability of forgings [3]. Due to the relatively high manufacturing costs of 42CrMo4 steel forgings, research is necessary to develop an alternative, cheaper steel showing high mechanical properties and maintaining good technological properties. Modern advanced steels for forgings should show a good combination of high strength, ductility and fracture resistance under different loading conditions [4,5,6]. Obtaining beneficial properties should be achieved at the lowest possible cost, which is related to minimizing the amount of alloying elements added into the steel and reduction of heat treatment operations applied after the forming process [7].

Medium-Mn steels containing retained austenite (RA) are promising novel materials for forging applications due to the ability to provide high strength and fracture resistance at lower cost, when compared to the 42CrMo4 type steel [8,9]. Multiphase steels containing RA are typically produced as hot-rolled or cold-rolled sheets [10,11,12]. In the available literature, there are a very limited number of reports on the application of this steel type for forgings. Sugimoto et al. [13] investigated the influence of forging parameters on the mechanical properties of a multiphase 0.42C-1.51Mn-1.47-Si-0.5Cr-0.48Al-0.2Mo-0.05Nb steel. The steel showed beneficial mechanical properties: YS~880 MPa, UTS~1390 MPa and good impact toughness~88 J. However, the necessity of applying multi-step cooling, microstructural heterogeneity and the challenging machinability of steels with the increased carbon content significantly limit their potential applications for forgings. Due to the increased Mn content (3–10 wt.%) providing high hardenability and a limited C content (below 0.3 wt.%) ensuring good machinability, medium-Mn steels could be better suited for forgings than other multiphase steels [8,9].

Despite the mentioned advantages, from an industrial point of view an investigation into the hot flow behavior of medium-Mn steels is crucial for their practical application. The Gleeble thermomechanical simulator allows precise determination of flow stresses at different temperatures and strain rates corresponding to the hot deformation conditions [14]. The hot compression test carried out using the Gleeble simulator is commonly used to characterize the hot working behavior of steels [15,16,17]. Luo et al. [14] investigated the compressive deformation behavior of 30Cr2Ni3MoV steel for forgings at a temperature range of 950–1150 °C at different strain rates. They used the obtained data for developing a model predicting thermal deformation behavior and microstructure evolution of the 30Cr2Ni3MoV steel during hot deformation. Sun et al. [18] reported that results of hot compression tests are a good basis for developing a material model of the microstructure evolution of steel with elevated Mn content (10 wt.%). Investigations on the optimal process parameters and hot working behavior of medium-Mn steels with lower Mn contents (3–5 wt.%) are still very limited and require further investigations. Numerical modelling based on finite element method (FEM) is an effective tool allowing the prediction of the material flow behavior, which can be used for designing and optimizing the forging process [19,20].

Due to the ability to provide high strength, ductility and fracture resistance at reduced production costs, the novel 0.17C-3.92Mn-1.02Si-0.53Al-0.22Mo-0.032Ti-0.069V steel was developed as a promising alternative to conventional 42CrMo4 steels used for forgings. However, its application in the industry requires investigations on its hot deformability. Therefore, the main objective of this study was to predict the deformation behavior of the investigated steel during continuous hot compression using the FEM analysis, providing a reference for further optimization of the press forging process of multiphase medium-Mn steels containing RA. The identification of material model parameters was performed based on the experimental results of the hot compression tests carried out using the Gleeble thermomechanical simulator.

## 2. Materials and Methods

### 2.1. Material

The experimental 0.17C-3.92Mn-1.02Si-0.53Al-0.22Mo-0.032Ti-0.069V steel was produced by vacuum induction melting in a PVA TePla VSG-100 furnace (PVA Industrial Vacuum Systems GmbH, Wettenberg, Germany). Then, the ingot with a mass of about 100 kg and dimensions of 100 × 100 mm was homogenized in an electric furnace (resistance heating) at 1200 °C for 2 h and then hot-rolled in a temperature range of 1200–900 °C to a thickness of about 22 mm with a deformation degree~25%, followed by air cooling to room temperature. The hot rolling process was performed using the LPS/B semi-industrial line located at Łukasiewicz Research Network—Upper Silesian Institute of Technology (Gliwice, Poland).

### 2.2. Gleeble Thermomechanical Simulations

**Figure 1:**
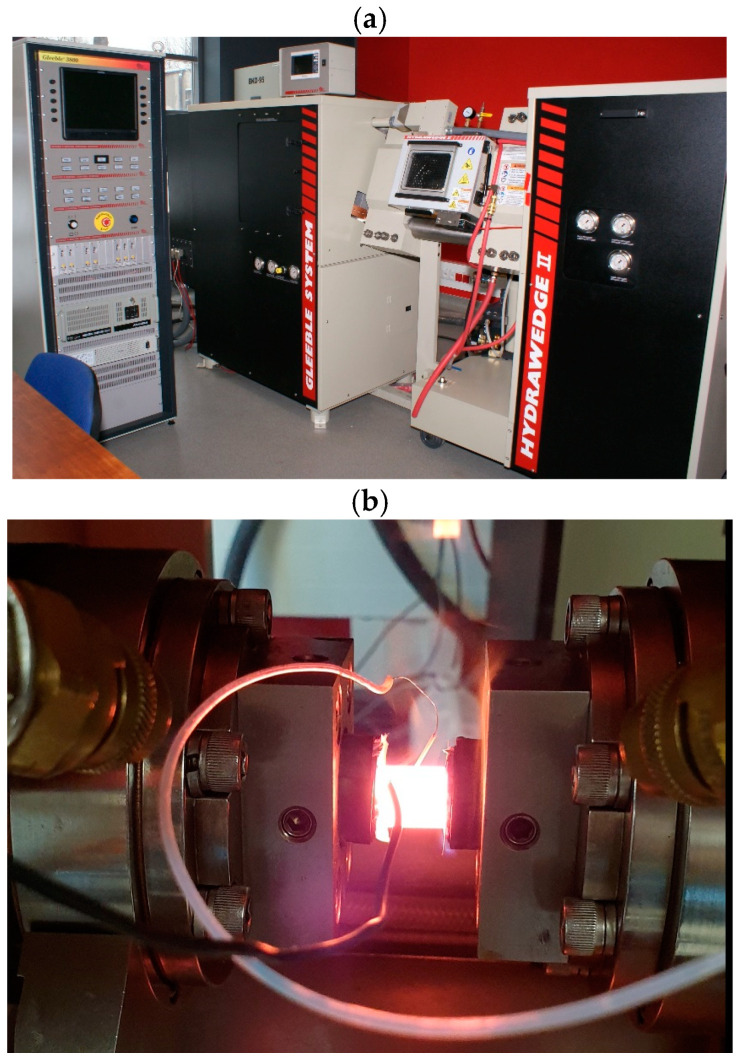
The Gleeble 3800 system equipped with a Hydrawedge Module (**a**) and an axisymmetric Gleeble sample during compression tests (**b**).

**Figure 2:**
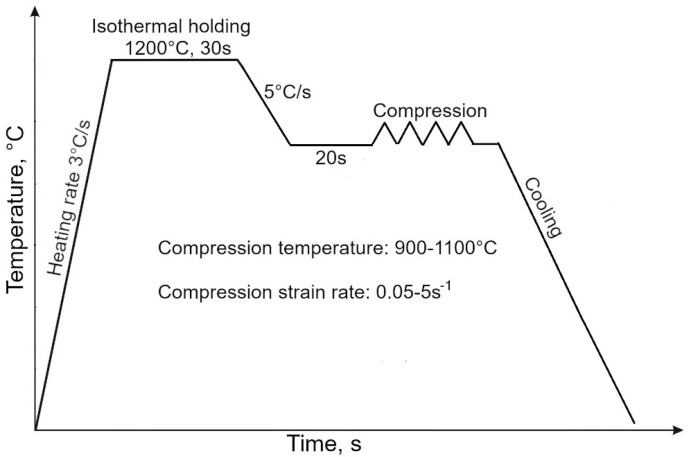
The scheme of continuous hot compression tests.

Cylindrical samples with a diameter of 10 mm and a length of 12 mm were machined from a hot-rolled 22 mm plate aligned with the rolling direction and used for hot compression tests performed in a Gleeble 3800 simulator (Dynamic Systems Inc., Poughkeepsie, NY, USA) using a Hydrawedge module. The strain rate during forging differs significantly depending on the equipment used, such as presses or drop hammers. Press forging is typically performed at low strain rates because the deformation is applied rather slowly and over a longer time. Depending on the specific type of press used, the strain rate is from 1 to 10 s^−1^. Drop hammer forging is associated with the rapid application of deformation resulting in significantly higher strain rates compared to press forging. Strain rates in drop hammer forging can exceed even 100 s^−1^ depending on the equipment used and the process settings. Simulating the conditions occurring during drop hammer forging requires experimental data obtained for significantly higher strain rates. Isothermal compression tests were carried out at the temperatures of 900, 1000 and 1100 °C at strain rates of 0.05, 0.5 and 5 s^−1^ to cover a wide range of parameters and including conditions occurring during press forging [14]. The samples were subjected to resistance heating at 1200 °C in a vacuum chamber for 30 s. Then, samples were cooled to a target deformation temperature and held for 20 s before compression. After a compression reduction corresponding to the true strain of 1, the samples were cooled to room temperature. Samples selected for microstructure observations and hardness measurements were water-cooled and the other samples were air-cooled. The Gleeble 3800 system equipped with a Hydrawedge Module and an axisymmetric Gleeble sample during compression tests are shown in Figure 1a and Figure 1b, respectively. The scheme presenting the details of the hot compression tests is shown in Figure 2.

### 2.3. Numerical Investigations

**Figure 3:**
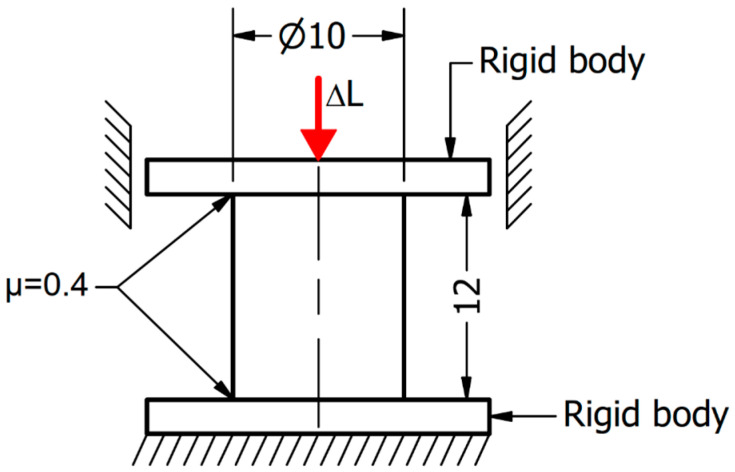
The scheme of the prepared numerical model.

Numerical research was conducted with the use of the FEM in the commercially available software LS-Dyna R.15 0.1 which, due to its possibilities, is widely used in many fields of science [21,22,23,24,25,26,27,28], as well as for metal forming analysis [29,30,31]. The prepared model consisted of three parts: a deformable sample with a diameter of 10 mm and a length L_0_ of 12 mm, and two anvils with a diameter of 20 mm. As the result of a discretization process, a volume of the sample was divided into 241,050 four-noded tetragonal finite elements with a length in the range of 0.15 to 0.25 mm. The stiffness of the anvils was much higher when compared to the investigated samples (Young’s modulus of the anvil material was 598 GPa); thus, they were treated as rigid bodies. Therefore, the surfaces representing anvils were divided into triangular shell elements. During the Gleeble tests, the temperature of the sample and the anvil was almost the same. Therefore, the heat transfer between the sample and the anvils was negligible. Thus, it was not taken into account. If the temperature of a sample is different than the temperature of the anvils, heat transfer between the sample and anvils should be taken into account [32]. One-way frictional contact was applied between the sample and the rigid anvils. A friction coefficient had been set as 0.4 [19]. All degrees of freedom had been locked for the fixed anvil. For the movable anvil, all degrees of freedom had been locked except translational movement through the sample axis direction. Uniaxial compression of the sample was reached due to the change in the movable anvil position. A strain (ε) value of 1 was assumed. Based on the equation (Equation (1)), the displacement of the anvil, which corresponded to the assumed strain, was calculated as ∆L = 7.6 mm. A scheme of the prepared model is presented in Figure 3.

(1) $$\varepsilon =\ln\left(\frac{L_{0}+∆L}{L_{0}}\right)$$

**Figure 4:**
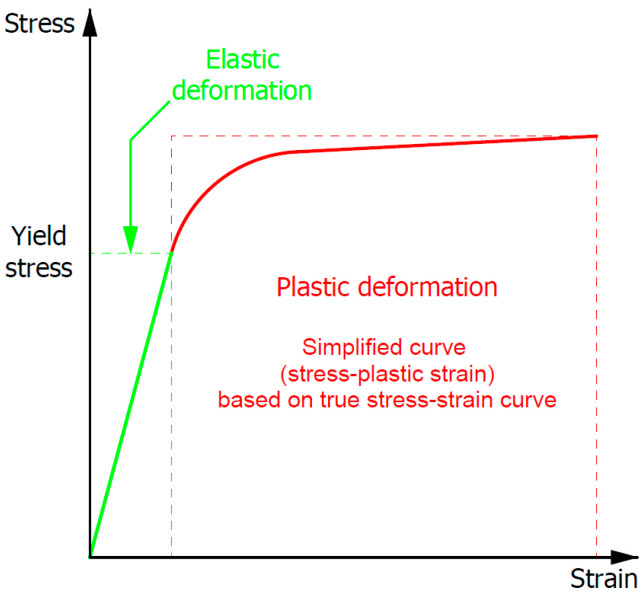
Schematic stress–strain curve of the selected material model.

An elastic–plastic material model was selected to describe the stress–strain dependence of the investigated samples. In the selected material model, deformation of the material was elastic as long as the stress did not reach a defined yield stress value. Then, the plastic deformation of the material was described by a multilinear, simplified stress–plastic strain curve. The curves were developed with a number of points that allowed for a good correlation of their course (in a whole range of assumed plastic strain) with curves obtained from Gleeble tests. Thermal strain as well as heating and cooling effects on the material properties’ changes were not taken into account during the numerical investigation, which was focused on the plastic strain distribution during the uniaxial compression of the sample. A schematic stress–strain curve of the selected material model is presented in Figure 4.

### 2.4. Hardness Measurements

Vickers hardness measurements were performed on the Gleeble samples after hot compression tests for experimental validation of the results obtained in FEM simulations. The hardness changes reflected the gradient distribution of plastic strain in the Gleeble samples. A microhardness tester FB-700 (Future-Tech Corp., Kawasaki, Japan) and a load of 1 kN were used. Samples after compression tests were cut in half and the hardness measurements were performed at vertical and horizontal axes of the sample along their geometric center. The measurements were repeated five times to obtain a mean value of hardness.

### 2.5. Microstructure Observations

The microstructure of the investigated steel after the physical compression tests using a Gleeble thermomechanical simulator was investigated using a Zeiss Observer Z1m light microscope (Oberkochen, Germany) to identify whether any changes in the microstructure were visible as a result of the gradient distribution of plastic strain in the Gleeble samples. Samples after the compression test were cut in half and embedded in a resin. Afterwards, the samples were ground using SiC paper up to 1200 grit, polished with a diamond paste and finally etched in 4% nital solution to reveal the microstructure.

## 3. Results and Discussion

### 3.1. Flow Behavior Analysis

Industrial trials aimed at designing processing strategies for newly developed steel grades are expensive and cause disruptions of production schedules. An alternative approach involves physical and numerical simulations of the deformation behavior of steel during the hot working process [2,14,15]. Parameters of the hot working process significantly affect the mechanical properties of medium-Mn steels [9,18,19] and therefore it is important to characterize their deformation behavior under conditions similar to those occurring during industrial processes. The Gleeble simulator allows a simulation of the forging process itself, taking into account primarily the basic process parameters such as deformation temperature, strain rate and strain value. Based on the experimental results, which are carried out on small samples, one can determine the structure and mechanical properties of the tested materials, but it must always be borne in mind that these are simplified experiments.

**Figure 5:**
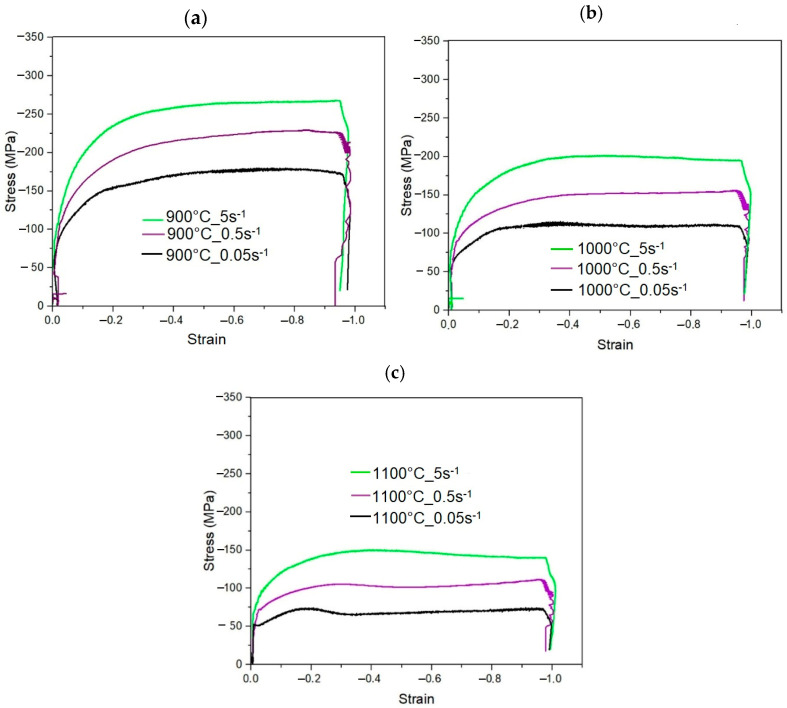
Stress–strain compression curves of 0.17C-3.92Mn-1.02Si-0.53Al-0.22Mo-0.032Ti-0.069V steel registered at different strain rates and temperatures: (**a**) 900 °C; (**b**) 1000 °C; (**c**) 1100 °C.

The flow behavior of the steel determined for various hot deformation conditions is especially important in designing and optimizing the hot forging process. The stress–strain curves of the investigated steel compressed at temperatures of 900, 1000 and 1100 °C and at strain rates of 0.05, 0.5 and 5 s^−1^ are shown in Figure 5. At the initial stage of deformation, the stress values significantly increase due to the formation of dislocations generating significant work hardening. Along with increasing strain, the rate of stress increase gradually decreases. At 900 °C (Figure 5a) and 1000 °C (Figure 5b), after reaching the plateau, flow stresses are at an almost constant level, which is related to the occurrence of a dynamic recovery process leading to dislocation annihilation resulting in the reduction of the work hardening rate [17]. Such a state is typical for the dynamic equilibrium between the dislocation generation and their annihilation due to thermally activated processes occurring in the microstructure [33,34]. As strain continues to accumulate, the effect of dynamic recovery becomes more significant and the rate of stress increase gradually slows down.

It was also observed that the stress values decrease with increasing deformation temperature and decreasing strain rate. This is due to the enhanced intensity of the dynamic recovery process controlling the flow stress of the investigated steel [35]. The elevated deformation temperatures promote grain boundary migration and reduce the resistance for dislocation slip enhancing softening effects [18]. Lower strain rates provide more time for the initiation of thermally activated processes and thus a lower work hardening rate was observed.

The initiation of dynamic recrystallization is only possible at 1100 °C, which is typical in such types of high-strength low-alloyed steels [18]. The strain levels necessary for dynamic recrystallization are relatively higher compared to steels containing 1–2 wt.% Mn [36,37]. A maximum peak stress was achieved for strain values of 0.18, 0.27 and 0.4 at strain rates of 0.05, 0.5 and 5 s^−1^, respectively. This behavior enables refining of the austenitic microstructure through dynamic recrystallization in the initial stages of forging, whereas a further grain refinement is only possible through partial static recrystallization between forging passes [14]. The limited range of recrystallization confirms the importance of applying Ti and V microalloying in forged products [33,34]. Both the registered flow stress and peak stress were higher when compared to the conventional steels containing 1–2 wt.% Mn [33,38].

### 3.2. Identification of Material Model Parameters

The results of hot compression tests performed in the Gleeble thermomechanical simulator were used for identifying material model parameters developed using FEM. In all analyzed cases, the Poisson’s ratio had been set as 0.3 [39,40]. Other elastic and plastic properties of the material model had been defined separately for each investigated sample. The Young’s modulus, yield stress and stress–plastic strain dependence had been determined based on true stress–strain curves obtained during hot compression tests carried out using the Gleeble simulator. Three measurements were performed for each test condition. The determined Young’s modulus and yield stress for each analyzed sample are presented in Table 1.

With the strain rate increase, the Young’s modulus and yield stress also increase. The opposite dependency is visible in the case of the temperature impact. The scope of material properties depends on the temperature applied. At higher temperatures, the changes in the Young’s modulus depend on the strain rate to a greater extent than at lower temperatures. The Young’s modulus varies from 3.0 to 3.4 GPa at 900 °C, from 2.0 to 2.9 GPa at 1000 °C and from 1.15 to 2.25 GPa at 1100 °C. This means a 13% increase at the lowest compression temperature and a 96% increase at 1100 °C. The change in the yield stress is more balanced with increasing temperature and strain rate (Table 1).

**Figure 6:**
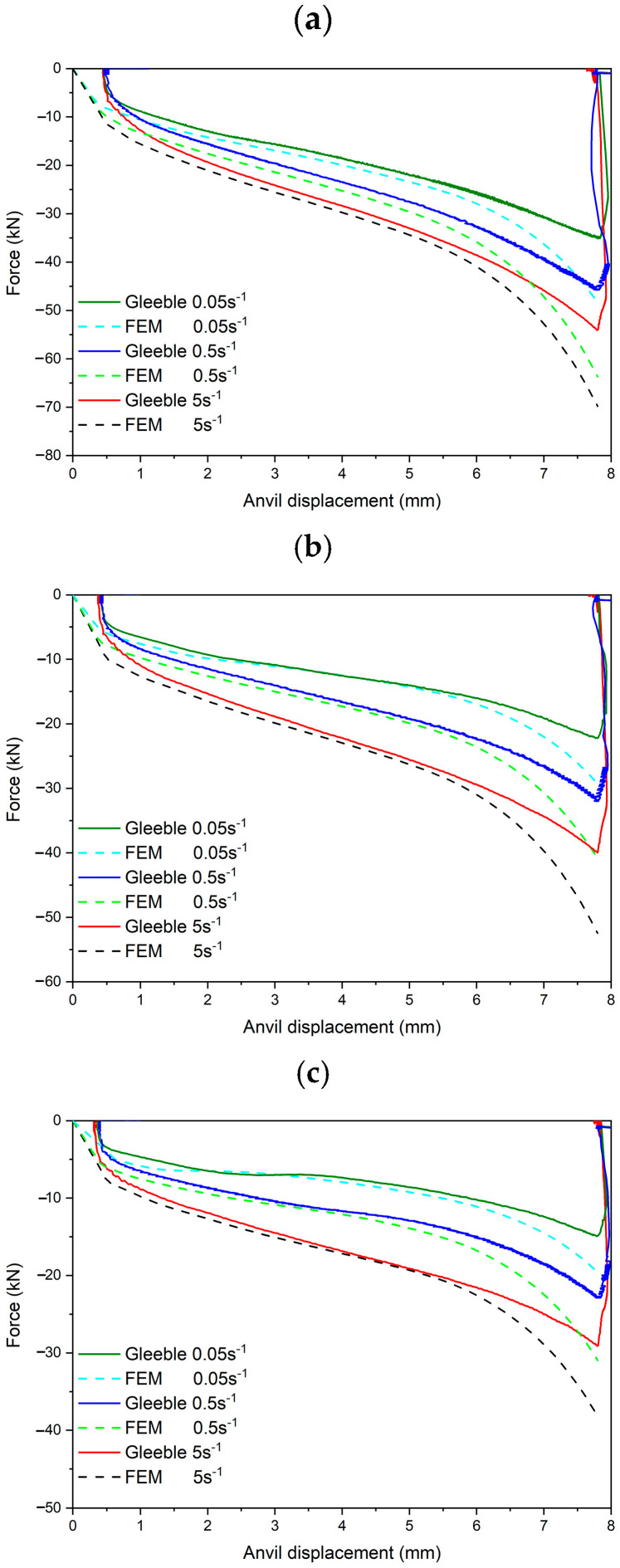
Dependences between the compression force of the movable anvil versus its displacement for the different temperatures of hot compression: (**a**) 900 °C; (**b**) 1000 °C; (**c**) 1100 °C.

The dependence between the compression force of the movable anvil versus its displacement under various test conditions (temperature and strain rate) is presented in Figure 6. Curves obtained from the experimental and numerical research are characterized by good compatibility. Only two stages of the curves look slightly different. The initial stage is related to the beginning of the test, when strain during the test is not measured perfectly. This effect is observed because samples in the Gleeble 3800 simulator in the Hydrawedge module are installed in a horizontal position and require the application of a small compressive force (about 100 kN) that will allow the sample to be held and the heating and annealing process to be stable during the test. This applied force allows the material to be deformed slightly (flowing), especially at high temperatures, i.e., during annealing at the austenitizing temperature of 1200 °C. Thus, before the final compression begins, the deformation value is zeroed and recalculated in relation to the real height of the sample.

**Figure 7:**
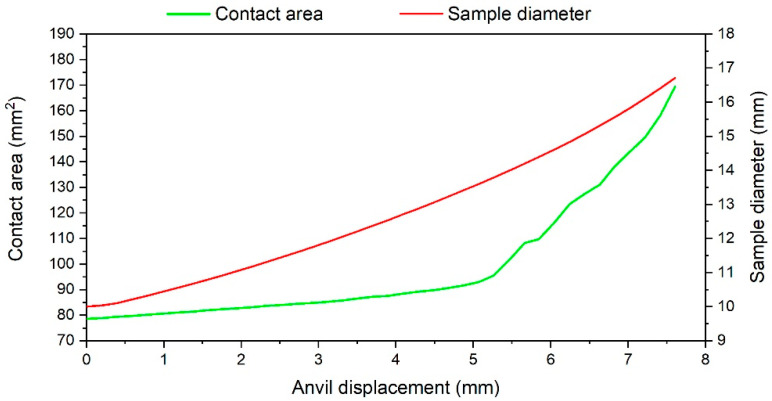
Change in the sample diameter and contact area during the compression test at 1000 °C and strain rate of 0.5 s^−1^.

The second stage starts at an anvil displacement of ca. 6 mm. It can be seen that the force in the numerical research starts increasing rapidly, which is not visible in the curves related to the experimental research. This phenomenon in the numerical research is related to the way in which the material model properties are obtained. The performed continuous compression test at the high temperature in the case of the higher plastic deformation of the sample leads to the barrel-shaped sample and non-uniform deformation across the entire cross section of the sample. Especially the diameter of the sample (measured in the middle of the sample high) and the contact area between the sample and the anvils are changed. An example of this change during the numerical research at 1000 °C and strain rate of 0.5 s^−1^ is presented in Figure 7.

As can be seen, the diameter of the sample increases constantly during the test. Moreover, an increase in the sample diameter is quicker in the case of the higher anvil displacement (higher plastic deformation of the sample). The sample diameter obtained as a result of the Gleeble test is very similar to the sample diameter obtained in numerical investigations; the difference is less than 1 mm, which confirms the correctness of the selected friction coefficient set as 0.4. The tendency in the case of the contact area is similar—it increases as the anvil displacement increases. However, a contact area starts to increase rapidly at an anvil displacement of ca. 5.5 mm. This is because of the high deformation degree of the sample. In the first part of this curve (up to ca. 5.5 mm), the increase in the contact area is due to an increase in the sample diameter. In the second part of the curve (above ca. 5.5 mm), a rapid contact area increase is caused by the material, which is located at the cylindrical part of the sample. Due to the high plastic deformation, the material which is initially located at the cylindrical part of the sample starts to contact with the anvils, causing the rapid increase in the contact area. A displacement value in which a contact area starts to increase rapidly (Figure 7) is very similar to the point at which a pressing force starts to increase rapidly in the case of the numerical research (Figure 6). Due to the significant change in the contact area between the sample and anvils, the frictional forces start to increase rapidly, which is reflected by an increase in the movable anvil pressing force. In the Gleeble test, this phenomenon is masked in the obtained stress–strain data (because measured stresses are related to a machine pressing force). Therefore, the use of the stress–strain data registered in the Gleeble test at high plastic strain as reference data in numerical calculations causes some discrepancies (e.g., in measured forces) above the strain, which corresponds to the rapid increase in the contact area during the uniaxial compression test (Figure 6). In the case of higher plastic strains, frictional forces are included in the calculations twice. The first time is in the stress–strain curve from the test and the second time is as frictional forces caused by the friction coefficient between the sample and anvils. Mean values of the pressing force error between the Gleeble test and the numerical investigation (Figure 6) in the range of the movable anvil displacement of 2–6 mm are presented in Table 2.

The convergence between the pressing force values and the characteristics of the curves is quite good in the case of the movable anvil (Figure 6). A maximum mean error in the range of 2–6 mm displacement is 8.8%, when the minimum is 1.8%, which means a strong correlation of results [41,42]. During the numerical investigation, the true stress–strain curves of the samples from the uniaxial compression tests were taken as a material data reference (Figure 5). A higher pressing force error was directly related to the shape of the Gleeble true stress–strain curves. What is the most important is that the convergence of the model is very good at the true strain up to 0.8, which is typically used in industrial applications. Besides the FEM approach, other modelling methods are used to predict the hot deformation behavior of high strength steels. Luo et al. [14] used cellular automaton models to predict the thermal deformation behavior of 30Cr2Ni3MoV steel for forging applications. They compared modeling results with the data obtained from hot compression tests performed using a Gleeble thermomechanical simulator. The flow curves were also used for developing grain size evolution models describing the kinetics of recrystallization process [18,43].

### 3.3. FEM Numerical Analysis

**Figure 8:**
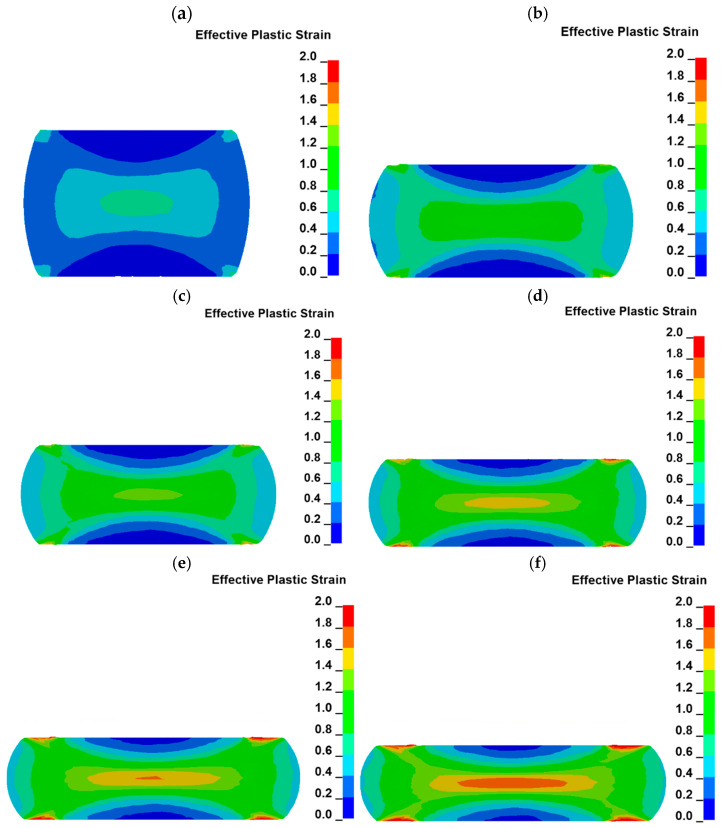
Color-coded maps of plastic strain distribution at the cross section of the sample (temperature of 1000 °C and strain rate of 0.5 s^−1^) at varied true strain: (**a**) 0.39, (**b**) 0.67, (**c**) 0.73 (**d**) 0.88, (**e**) 0.96, (**f**) 1.

The distribution of plastic strain in the material is very important during forging. Some examples of color-coded maps of the plastic deformation distribution during uniaxial compression at the cross section passing through the axis of the sample are presented in Figure 8.

An increase in the plastic strain distribution during uniaxial compression is similar to the letter “X” [42]. Such plastic distribution is registered during the whole test. As can be seen in the presented cross sections, plastic strain starts to increase in the geometrical center of the sample (plastic strain in a range of ca. 1.65–1.75) as well as in the areas near the sample edges (plastic strain in a range of ca. 1.8–2.3), which are the boundaries of the sample and have contact with the anvils. In Figure 8a, the maximum plastic strain is located in the geometrical center of the sample. In all other cross sections, the maximum strain is located in areas near the sample edges connected with anvils. This difference is related to the compression rate of the sample. As the compression is not high enough, as in Figure 8a, the plastic strain increases firstly in the geometrical center of the sample. The plastic strain starts to be higher at the areas near the edges of the sample than at its geometrical center, while the contact area between the sample and anvils starts to increase rapidly (Figure 7). The higher plastic strain distribution in areas located near the edges of the sample is related to the frictional force between the sample and the anvils. The occurrence of friction forces between the sample and the anvils causes also the sample to become a barrel after the compression. The distribution of the plastic strain is in accordance with reports described by other researchers [42,44].

**Figure 9:**
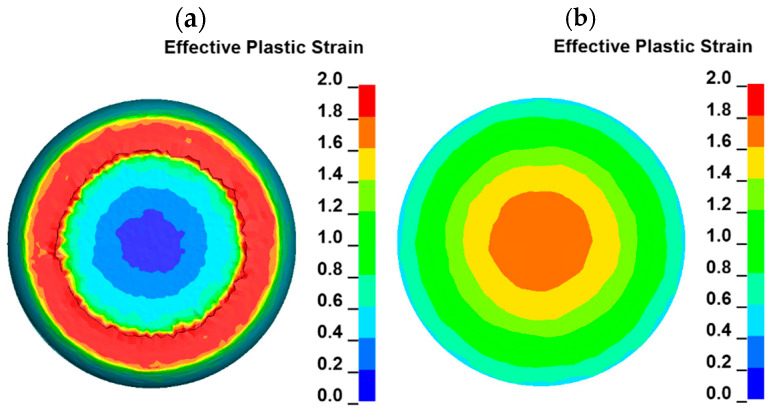
Color-coded maps of plastic strain distribution (temperature of 1000 °C and strain rate of 0.5 s^−1^): (**a**) a view from the top of the sample, (**b**) a view at the middle of the compressed sample height.

Areas of high plastic strain (in the geometrical center of the sample and near the sample edges) are connected by some kind of bridges at which the plastic strain is lower than in the mentioned areas, but it is also higher compared to the rest of the sample cross section. The lowest plastic strain, in a range of ca. 0.1–0.2, can be observed in the areas which are located in the axis of the sample and have contact with the anvils. They are areas of high resistance to plastic deformation because of the geometry of the sample and anvils. Generally, the material in this area has no direction in which it could easily flow during the uniaxial compression [14,18]. Thus, the plastic strain distribution in the volume of the sample is very complex, which can be seen in the cross sections parallel to the sample axis, as well as in the cross section perpendicular to the sample axis and located at the middle of its height after compression. Plastic strain distribution in this cross section, as well as at the top of the sample, is presented in Figure 9.

**Figure 10:**
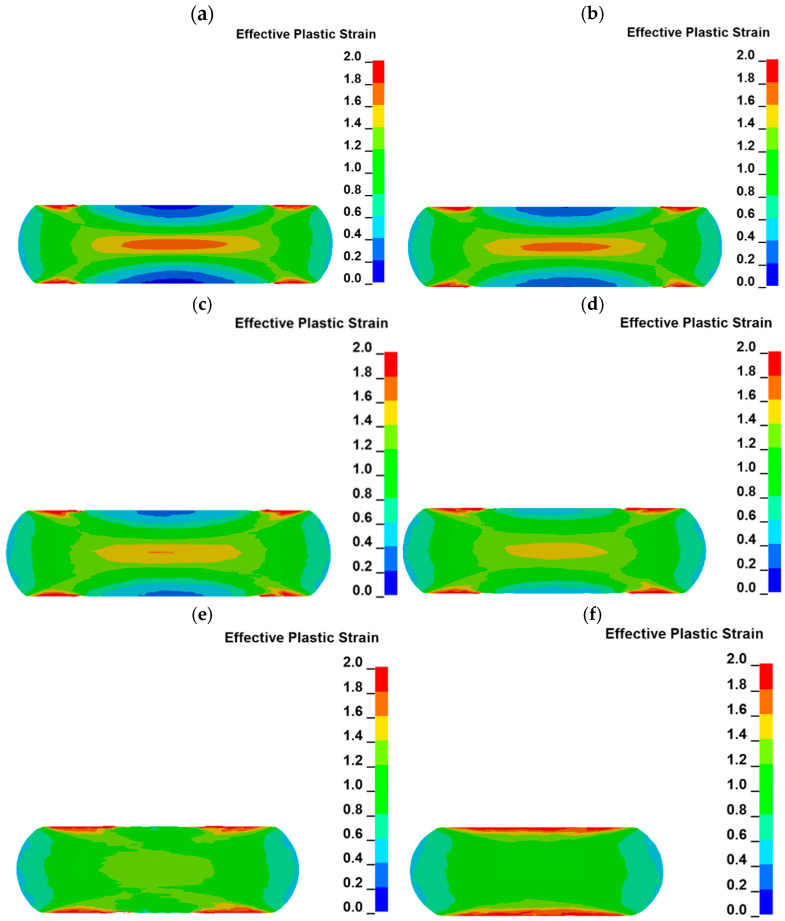
Colored-coded maps of plastic strain distribution in the sample (temperature of 1000 °C and strain rate of 0.5 s^−1^) at cross sections located at various distances from the sample axis: (**a**) 1 mm, (**b**) 2 mm, (**c**) 3 mm, (**d**) 4 mm, (**e**) 5 mm, (**f**) 6 mm.

The plastic strain at the top of the sample increases as the radius of the sample increases (Figure 9a). High values of plastic strain are located in the area which is moving through the anvil during sample compression. During material movement, the frictional force occurs, so the amount of plastic strain is the highest in this area. The area with the lowest plastic strain is located in the center of the sample. The situation is opposite in the case of the cross section of the sample which is presented in Figure 9b. The highest strain is observed in the area located in the center of the sample. As the distance from the axis of the sample increases, the plastic strain decreases. The analysis of the plastic strain distribution presented in Figure 9 confirms the conclusions obtained from the cross sections presented in Figure 8. The distribution of the plastic strain also varies in the planes which are parallel to the sample axis but located at various distances. Such plastic strain distribution is presented in Figure 10.

Characteristic plastic strain distribution in the shape of the letter X starts to be less visible as the distance from the axis of the sample increases. The area with the highest strain is located near the sample edges, which have contact with the anvils in all the presented cross sections. These areas grow as the distance from the axis of the sample increases. Plastic strain distribution is the most uniform in the sample volume at the cross section located at the highest distance from the axis of the sample.

### 3.4. Hardness Measurements and Microstructure Observations

**Figure 11:**
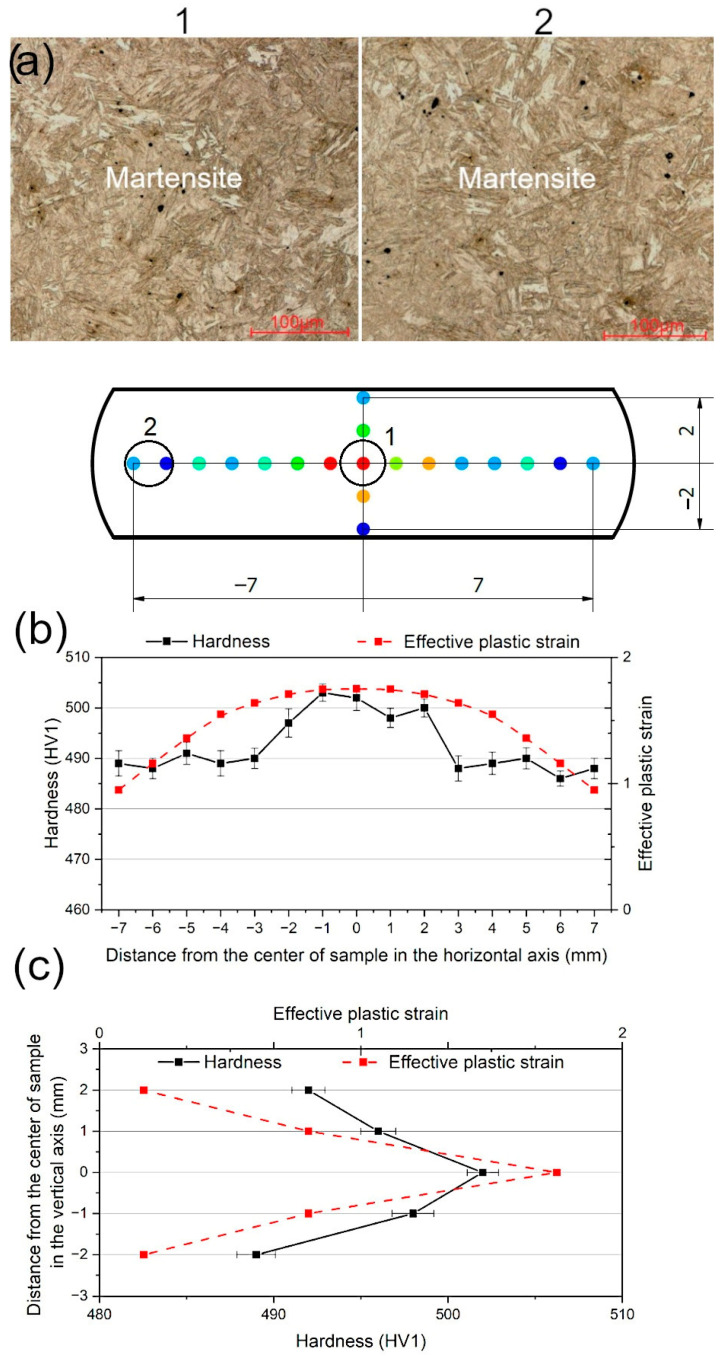
The hardness measurement profiles and corresponding effective plastic strain values together with microstructures of the Gleeble sample compressed at 1000 °C at a strain rate of 0.5 s^−1^ (**a**) collected along the horizontal line in the middle of the sample’s height (**b**) and along the sample’s axis (**c**).

The FEM results were focused on the plastic strain distribution in a sample volume during compression. The developed numerical model did not take into account heating and cooling. The performed experimental and numerical investigations were focused on the hot deformation behavior of the investigated steel instead of any influence of the cooling rate on the microstructure or hardness. In our numerical research, the test conditions such as temperature and strain rate were taken into account by using the experimental stress–strain curves obtained from the Gleeble test results for each investigated case separately. The results of the FEM analysis were experimentally validated by hardness measurements. The measurements were performed along the vertical and horizontal axes of the sample at its geometric center to show the gradient distribution of plastic strain (Figure 11a). Results of hardness measurements reflect the changes in the microstructure of the steel during compression tests. The hardness measurements performed in the vertical and horizontal axes of the sample after the hot compression test carried out at 1000 °C and a strain rate of 0.5 s^−1^ are shown in Figure 11b,c. The obtained results show different hardness values with various distances from the central zone of the sample. Interestingly, the relatively rapid increase in hardness was measured at the distance of 3 mm from the deformed sample axis (Figure 11b) and in the middle of the sample’s height (Figure 11c), which corresponds to the highest strain ranges identified in the strain distribution maps (Figure 8f). The results of the hardness measurements (Figure 11b,c) show good correlation with the values of plastic strain. The highest hardness (500–503 HV1) measured along the horizontal axis of the sample was associated with the highest values of effective plastic strain~1.65–1.75 (Figure 11b). The highest hardness (502 HV1) measured along the vertical axis of the sample aligned well with the highest value of effective plastic strain~1.75 (Figure 11c). The course of the curves corresponding to the effective plastic strain reflects the trend of the hardness measurements in Figure 11b,c. The rest of the sample shows the almost constant hardness in a range of 486–491 HV1.

Although the metallographic investigations were taken in the different zones of the sample corresponding to the significantly different strain values, i.e., 1.65–1.75 for point 1 and 0.95–1.05 for point 2, the sample was characterized by the same martensitic microstructure (Figure 11a). Due to the increased Mn addition (3.92 wt.%), the investigated steel showed high hardenability. Thus, the fully martensitic microstructure showing high hardness can be obtained in a wide range of cooling rates. A reduction in hardness can be observed only at very slow cooling rates of about 0.1–0.05 °C/s due to the self-tempering effect of martensite [45]. However, the different hardness of martensitic microstructures indicates indirectly a differentiated grain size and/or dislocation density [33,36] in differently strained zones of the samples but it will be investigated in more detail elsewhere.

## 4. Conclusions

The hot deformation behavior of novel 0.17C-3.92Mn-1.02Si-0.53Al-0.22Mo-0.032Ti-0.069V steel intended for advanced high-strength automotive forgings was characterized using physical and numerical approaches. Based on the obtained results, the following conclusions can be drawn:

- The hot flow behavior of the investigated steel depends on the deformation temperature and strain rate. The stress values decrease with increasing deformation temperature and decreasing strain rate due to the enhanced intensity of dynamic recovery. The dynamic recrystallization controls the hot working behavior only at 1100 °C. The Young’s modulus and yield stress highly depend on the test temperature and strain rate. With the strain rate increase, the Young’s modulus and yield stress also increase. The opposite dependency occurs for temperature changes. At 1100 °C, the changes in the Young’s modulus depend on the strain rate to a greater extent than at lower temperatures. At the lowest analyzed temperature of 900 °C, the Young’s modulus increases about 13%, and at 1100 °C it increases about 96%, with an increasing strain rate from 0.05 to 5 s^−1^.
- Plastic strain distribution maps provide a lot of valuable information on the changes occurring in the sample volume during the uniaxial compression. The highest plastic strain (ca. 1.8–2.3) is observed in small areas located at the edges of the sample, which are under frictional contact with anvils. Another area of the sample volume characterized by high plastic strain (ca. 1.65–1.75) is located in the geometrical center of the sample. The lowest plastic strain in a range of ca. 0.1–0.2 can be observed in the areas which are located on the axis of the sample and have contact with the anvils. Thus, the plastic strain distribution in the sample cross section is similar to the letter “X”.
- Results of the FEM analysis show good compatibility with the experimental results. The rapid increase in hardness was measured at the distance of 3 mm from the sample axis, which corresponds to the highest strain ranges identified in the strain distribution maps. The highest hardness values in a range from 500 HV1 to 503 HV1 were noted in the zones experiencing the true strain in a range of 1.65–1.75. Regardless of the microstructure observation zone, the samples show fully martensitic microstructure due to high hardenability provided by the increased Mn addition.

## Figures and Tables

**Table 1:** Material’s properties determined based on hot compression tests performed using the Gleeble thermomechanical simulator.

Temperature [°C]	900			1000			1100		
Strain rate [s^−1^]	0.05	0.5	5	0.05	0.5	5	0.05	0.5	5
Young’s modulus [GPa]	3.0	3.2	3.4	2.0	2.7	2.9	1.15	2.0	2.25
Yield stress [MPa]	82	115	140	67	91	122	63	72	97

**Table 2:** The force error between the Gleeble experiment and the numerical investigation in the range of 2–6 mm displacement.

Temperature [°C]	900			1000			1100		
Strain rate [s^−1^]	0.05	0.5	5	0.05	0.5	5	0.05	0.5	5
Pressing force error [%]	7.4	8.5	5.2	1.8	5.3	4.0	5.7	6.2	8.8

## Data Availability

The original contributions presented in this study are included in the article. Further inquiries can be directed to the corresponding author.
